# Dermatoglyphic features in patients with multiple sclerosis

**Published:** 2014-10

**Authors:** Vedat Sabanciogullari, Seyda Cevik, Kezban Karacan, Ertugrul Bolayir, Mehmet Cimen

**Affiliations:** *From the Departments of Anatomy (Sabanciogullari, Cimen) and Neurology (Cevik, Bolayir), School of Medicine, Cumhuriyet University, Sivas, and the Department of Anatomy (Karacan), School of Medicine, Sakarya University, Sakarya, Turkey*

## Abstract

**Objective:**

To examine dermatoglyphic features to clarify implicated genetic predisposition in the etiology of multiple sclerosis (MS).

**Methods:**

The study was conducted between January and December 2013 in the Departments of Anatomy, and Neurology, Cumhuriyet University School of Medicine, Sivas, Turkey. The dermatoglyphic data of 61 patients, and a control group consisting of 62 healthy adults obtained with a digital scanner were transferred to a computer environment. The ImageJ program was used, and atd, dat, adt angles, a-b ridge count, sample types of all fingers, and ridge counts were calculated.

**Results:**

In both hands of the patients with MS, the a-b ridge count and ridge counts in all fingers increased, and the differences in these values were statistically significant. There was also a statistically significant increase in the dat angle in both hands of the MS patients. On the contrary, there was no statistically significant difference between the groups in terms of dermal ridge samples, and the most frequent sample in both groups was the ulnar loop.

**Conclusions:**

Aberrations in the distribution of dermatoglyphic samples support the genetic predisposition in MS etiology. Multiple sclerosis susceptible individuals may be determined by analyzing dermatoglyphic samples.

Multiple sclerosis (MS) is the most frequent neurological disease of the CNS that causes permanent disability in young adults. The disease is characterized with chronic inflammation and demyelination of the CNS. The etiology of the disease is still not fully understood. However, in epidemiologic and genetic studies, heredity and environmental factors are suspected in the disease pathogenesis.^1^-^3^ Dermatoglyphics are specific patterns made by the epidermis ridges on the fingertips, palms, and soles. They are formed in the ectoderm between the eleventh and twenty-fourth weeks of intrauterine life and remain unchanged. In the same period, the CNS begins to occur from the ectoderm. During this period, any genetic disorder that affects the nervous system also leads to changes in dermatoglyphs. Thus, aberrations observed in dermatoglyphic samples indicate a hereditary disorder in the intrauterine period.^4^,^5^ In dermatoglyphic studies, the dermatoglyphic features of the society gains importance. There is a lack of research studying the dermatoglyphic features of MS patients. Therefore, to explore the genetic predisposition of MS etiology, the fingertip, and palm dermatoglyphic samples of patients with MS were compared with a control group and reviewed with the relevant literature.

## Methods

**Figure 1:**
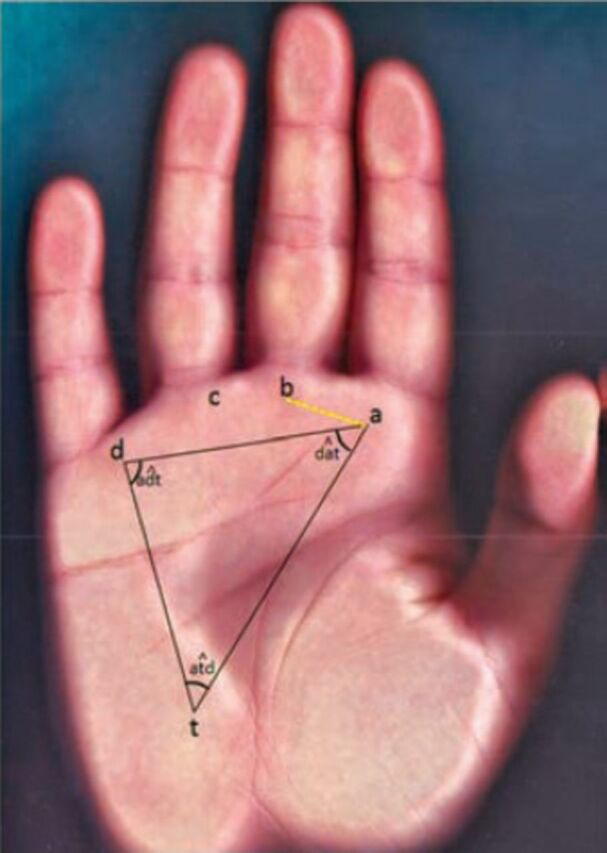
The palmar dermatoglyphic samples. The triradius that are located close to the each finger proximal were shown a, b, c, d letters starting from index finger. Axial triradius was shown by the letter t. Yellow line, between a and b triradius was used to determine the number of dermal lines. The angles atd, adt, and dat: Angles formed by connecting lines from digital triradii ‘a’ and ‘d’ to axial triradius ‘t’ and connecting line from digital triradius ‘a’ to digital triradius ‘d’.

**Figure 2:**
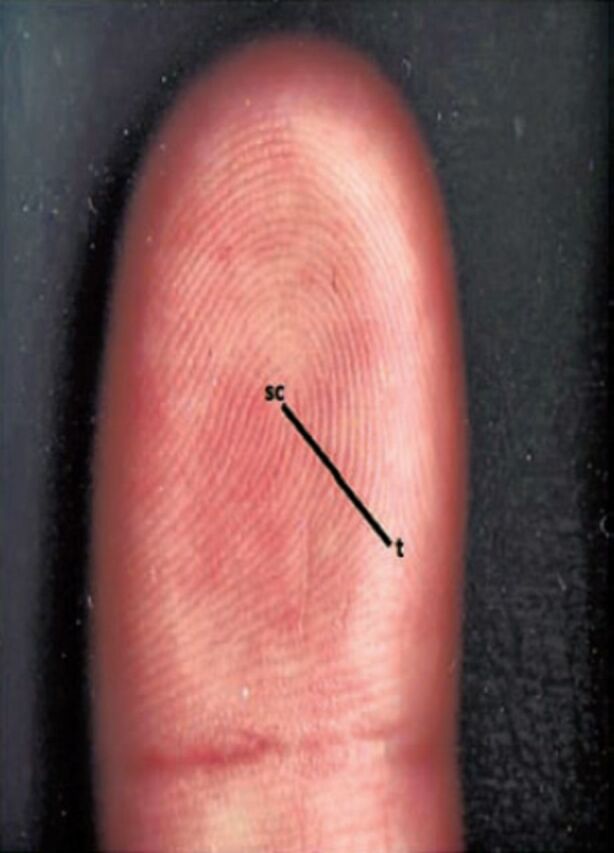
Calculation of the number of lines on the fingertip dermal samples. A vertical line is drawn to the most distant triradius from the center of the dermal sample. The number of dermal lines that intersect with this vertical line are counted and the number of lines in the sample is obtained. Sc - sample center, t - triradius

The study was conducted between January and December 2013 at the Departments of Anatomy and Neurology, Cumhuriyet University School of Medicine, Sivas, Turkey. The Human Ethics Committee of Sivas Province approved the study. Sixty-one patients with no other disease but MS were included in the study after giving their informed consent. Patients with burns on the fingers and palm of the hand, dermatologic disease, and hand deformities were excluded from the study. The average age of the patients was 35.21±10.42 (between 18-56 years old), 41 of whom were female, and 20 were male. The control group was comprised of 62 volunteers with no personal or family history of MS or other neurological disease. The average age of the individuals in the control group was 22.92±7.34 (between 18-50 years old), 35 of whom were female, and 27 were male. Dermatoglyphic samples were obtained using a digital scanner (CanoScan LIDE 60, Canon, Beijing, China). During the sampling, the palm was touching the scanner screen, the thumb was at approximately 30-40 degrees, and the other fingers were placed in 10-15 degrees abduction. Through scanning, 4 colored images with 300 dpi-resolution for each patient were recorded. Via images saved in jpg format, and by using the ImageJ program (NIH, Bethesda, MD, USA), fingertip sample types, fingertip ridge counts, a-b ridge count, atd, dat, and adt angles were determined (**Figures 1 & 2**). Fingertip sample types were evaluated, according to Cummins and Midlo classification,^6^ as whorl, ulnar loop, radial loop, and arch. The ridge counts except for arch were determined by counting the ridges between the sample center and the triradius (**Figure 2**). In the samples with more than one triradius, the side that had more ridge counts was evaluated. In the samples of arch, the ridge count was accepted as zero. The ridge counts in all fingers were gathered, and a total ridge count (TRC) was obtained. The axial triradius ‘t’ emerges from the combinations of ridge bundles coming from 3 different directions between the thenar and hypothenar parts in the palm by making an angle of 120 degrees between each other.

The collected data were analyzed using the Statistical Package for Social Sciences version 15 (SPSS Inc., Chicago, IL, USA). The T-test, Mann-Whitney U, and chi-square test was used in the statistical evaluation. *P*-values below 0.05 were considered significant.

## Results

There was a statistically significant increase in the a-b ridge count, total ridge count, and ridge count on all fingertips on both hands of patients with MS. Also, the measured dat angle of both hands was higher in patients with MS compared with healthy controls (*p*<0.05). The atd angle in the right hand of patients was lower than in healthy controls (*p*=0.001) (**Table 1**). The ulnar loop was observed as the most frequent sample type in both patients and healthy controls on the fingertips of the right hand. The radial loop was the least common sample in both groups (**Table 2**). The ulnar loop was the most frequently observed sample type in both patients and healthy controls on the fingertips of the left hands. In contrast, the radial loop was significantly higher in patients (*p*<0.05) (**Table 3**). There was an increase in both hand a-b ridge counts, total ridge counts, count of fingertips except the index finger of the right hand in female patients diagnosed with MS when compared with female controls (*p*<0.05). The atd angle in the right hand of female patients was lower than in healthy female controls (*p*=0.000) (**Table 4**). In both hands, total ridge counts, a-b ridge counts, and the dat angle of male patients was higher when compared with controls (*p*<0.05). The ridge count of fingertips increased in the right hand thumb, ring, and little fingers of male patients (*p*<0.05). The ridge count of fingertips increased in the left hand middle, ring, and little fingers of male patients (*p*<0.05) (**Table 5**).

**Table 1:** Comparison of the numbers and angles of fingertips and palmar lines in the control group and patients with multiple sclerosis.

Variable	Patient group (n=61)	Control group (n=62)	t-test	*P*-value	Patient group (n=61)	Control group (n=62)	t-test	*P*-value
	Right hand mean±SD	Right hand mean±SD			Left hand mean±SD	Left hand mean±SD		
Thumb	19.22±3.43	14.58±4.73	6.246	0.000	17.98±5.31	14.16±4.43	4.328	0.000
Index finger	14.95±7.75	12.41±5.97	2.026	0.045	14.52±7.44	11.74±5.81	2.307	0.023
Middle finger	16.29±6.76	12.08±6.15	3.611	0.000	15.40±6.87	11.54±6.15	3.280	0.001
Ring finger	17.88±4.45	13.03±5.42	5.426	0.000	17.78±4.58	12.79±5.85	5.276	0.000
Little finger	16.01±3.89	12.85±4.49	4.171	0.000	16.78±2.79	13.11±4.12	5.795	0.000
Total ridge count	84.57±18.78	64.27±0.23	5.461	0.000	82.24±18.58	63.32±21.39	5.240	0.000
atd angle (degrees)	39.61±4.07	42.34±4.76	-3.421	0.001	41.47±4.31	41.23±4.93	0.288	0.774
dat angle (degrees)	63.53±4.26	59.21±5.76	4.728	0.000	63.56±4.64	59.33±6.28	4.248	0.000
adt angle (degrees)	77.32±4.63	78.09±4.82	-0.893	0.374	75.53±4.74	79.02±5.36	-3.823	0.000
a-b ridge count	43.52±3.52	35.15±6.50	8.896	0.000	43.18±3.74	35.65±6.09	8.271	0.000

atd angle - angle formed by connecting lines from digital triradii ‘a’ and ‘d’ to axial triradius ‘t’, dat angle - angle formed by connecting lines from triradii ‘d’ and ‘t’ to triradius ‘a’, adt angle - angle formed by connecting lines from triradii ‘a’ and ‘t’ to triradius ‘d’, a - b ridge count - number of ridges between the triradius ‘a’ and triradius ‘b’

**Table 2:** The distribution of dermal samples in the right hand fingertips of patients with multiple sclerosis and the control group.

Variable	Patient group (n=61)				Control group (n=62)			
	Whorl	Ulnar Loop	Radial Loop	Arch	Whorl	Ulnar Loop	Radial Loop	Arch
Thumb	40	20	1	0	36	24	0	2
Index finger	24	20	6	11	32	14	8	8
Middle finger	10	41	4	6	15	36	2	9
Ring finger	32	26	1	2	35	21	1	5
Little finger	13	42	3	3	15	45	0	2
Total (%)*	119 (39.1%)	149 (48.8%)	15 (4.9%)	22 (7.2%)	133 (42.9%)	140 (45.2%)	11 (3.5%)	26 (8.4%)

* A percentage of the total number of dermal samples for each group individually

**Table 3:** The distribution of dermal samples in the left hand fingertips of patients with multiple sclerosis and the control group.

Variable	Patient group (n=61)				Control group (n=62)			
	Whorl	Ulnar Loop	Radial Loop	Arch	Whorl	Ulnar Loop	Radial Loop	Arch
Thumb	31	23	3	4	34	26	0	2
Index finger	23	20	9	9	30	15	10	7
Middle finger	13	35	5	8	17	34	1	10
Ring finger	22	33	2	4	23	33	0	6
Little finger	7	48	6	0	10	50	0	2
Total (%)*	96 (31.5%)	159 (52.1%)	25 (8.2%)	25 (8.2%)	114 (36.8%)	158 (51%)	11 (3.5%)	27 (8.7%)

* A percentage of the total number of dermal samples for each group individually

**Table 4:** The fingertips, palm ridge count, and palmar angles of females in patients with multiple sclerosis and the control group.

Variable	Patient group (n=41)	Control group (n=35)	t-test	*P*-value	Patient group (n=41)	Control group (n=35)	t-test	*P*-value
	Right hand mean±SD	Right hand mean±SD			Left hand mean±SD	Left hand mean±SD		
Thumb	19.31±3.18	13.97±4.54	5.841	0.000	18.46±4.45	13.40±5.01	4.620	0.000
Index finger	15.26±7.01	12.71±6.26	1.677	0.098	15.07±6.96	10.94±6.63	2.644	0.010
Middle finger	16.58±5.39	12.45±6.69	2.926	0.005	15.14±7.13	10.91±6.85	2.633	0.010
Ring finger	17.92±2.89	13.68±5.52	4.085	0.000	17.80±4.36	13.45±5.81	3.637	0.001
Little finger	15.63±4.36	12.68±5.10	2.682	0.009	16.75±2.83	13.25±4.28	4.121	0.000
Total ridge count	85.04±16.50	64.57±25.01	4.135	0.000	83.14±16.44	61.94±23.22	4.520	0.000
atd angle (degrees)	39.23±3.73	43.02±4.82	-3.789	0.000	41.83±3.56	42.11±4.72	-0.287	0.775
dat angle (degrees)	63.81±3.87	59.39 ± 6.59	3.482	0.001	63.62±4.04	58.84±6.77	3.658	0.001
adt angle (degrees)	77.44±4.97	77.14±5.22	0.256	0.799	74.93±4.70	78.83±6.24	-3.032	0.004
a-b ridge count	42.95±3.59	36.57±7.32	4.694	0.000	43.56±3.17	36.97±6.63	5.378	0.000

atd angle - angle formed by connecting lines from digital triradii ‘a’ and ‘d’ to axial triradius ‘t’, dat angle - angle formed by connecting lines from triradii ‘d’ and ‘t’ to triradius ‘a’, adt angle - angle formed by connecting lines from triradii ‘a’ and ‘t’ to triradius ‘d’, a - b ridge count - number of ridges between the triradius ‘a’ and triradius ‘b’

**Table 5:** The fingertips, palm ridge count, and palmar angles of males in patients with multiple sclerosis and the control group.

Variable	Patient group (n=20)	Control group (n=27)	Mann Whitney U (Z-)	*P*-value	Patient group (n=20)	Control group (n=27)	Mann Whitney U (Z-)	*P*-value
	Right hand mean±SD	Right hand mean±SD			Left hand mean±SD	Left hand mean±SD		
Thumb	19.05±3.97	15.37±4.93	2.828	0.007	17.00±6.77	15.14±3.39	1.123	0.272
Index finger	14.30±9.25	12.03±5.68	0.967	0.341	13.40±8.43	12.77±4.45	0.300	0.766
Middle finger	15.70±9.09	11.59±5.47	1.794	0.083	15.95±6.46	12.37±5.11	2.048	0.048
Ring finger	17.80±6.70	12.18±5.26	3.101	0.004	17.75±5.13	11.92±5.89	3.606	0.001
Little finger	16.80±2.60	13.07±3.64	4.083	0.000	16.85±2.77	12.92±3.97	3.985	0.000
Total ridge count	83.60±23.22	63.88±18.70	3.119	0.004	80.40±22.72	65.11±19.03	2.441	0.020
atd angle (degrees)	40.40±4.70	41.46±4.61	-0.771	0.445	40.75±5.59	40.10±5.06	0.409	0.685
dat angle (degrees)	62.97±5.03	58.98±4.57	2.786	0.008	63.43±5.80	59.97±5.64	2.045	0.047
adt angle (degrees)	77.08±3.94	79.31±4.02	-1.898	0.065	76.75±4.70	79.27±4.07	-1.915	0.063
a-b ridge count	44.70±3.14	33.30±4.79	9.827	0.000	42.40±4.71	33.93±4.93	5.973	0.000

atd angle - angle formed by connecting lines from digital triradii ‘a’ and ‘d’ to axial triradius ‘t’, dat angle - angle formed by connecting lines from triradii ‘d’ and ‘t’ to triradius ‘a’, adt angle - angle formed by connecting lines from triradii ‘a’ and ‘t’ to triradius ‘d’, a - b ridge count - number of ridges between the triradius ‘a’ and triradius ‘b’

## Discussion

When compared to the healthy control group, the a-b ridge count and total ridge count were prominently higher in both right and left hands of the female and male patients with MS in our study group. On the contrary, Supe et al^7^ reported that the ridge counts in the palms and fingertips of patients with MS were lower than the control group. Also, they found that the atd angle in both genders was lower in patients with MS. In our measurements, the values of the atd angle were also low, but these values showed a statistically significant difference in only the right hands of female patients with MS. The dat angle in both hands of both female and male patients was higher than that of the control group. Floris et al^8^ studied the dermatoglyphic asymmetry and differences in frequent diseases such as diabetes mellitus, hypertension, and MS. For this purpose, they analyzed the sample types in fingertips of 469 patients, and found that dermatoglyphic differentiation, in MS patients especially, was apparent.

Research studying the distribution of dermatoglyphic samples in MS patients is limited. There are studies reporting that dermatoglyphic analyses can be used as an adjuvant method in diagnosis, prognosis, and treatment of several diseases such as schizophrenia, Down syndrome, autism, various cancers, idiopathic epilepsy, and congenital heart diseases in which genetic predisposition plays a role in their etiologies.^9^-^14^ Fearon et al^9^ reported that the a-b ridge counts decreases in the patients with schizophrenia. They also claimed that individuals whose a-b ridge counts are relatively low may have a high risk for schizophrenia. Milicic et al,^10^ as a result of family study, stated that ridge count, a-b ridge count, and atd angle in the fourth and fifth fingers of autistic male patients’ of both hands were lower than those of a healthy control group, and this result was statistically significant. Sabanciogullari et al^13^,^14^ found that, in patients with panic disorder and idiopathic epilepsy, a-b ridge count increased, and fingertip whorl samples were higher in idiopathic epilepsy than the normal population. Sanches Cascos^11^ indicated that whorl samples are frequently seen in aortic stenosis, aortic coarctation, and Fallot tetralogy, arch type samples are mostly seen in patients with pulmonary stenosis, radial loops are the most rarely seen loop and they can be seen in patients with septal defect slightly more often, while ulnar loops are the most frequently seen sample in both groups. Chintamani et al^12^ reported that the average ridge count of patients with breast cancer was lower in both the right and left hand than that of the control group. They also stated that there was an increase in whorl samples on fingers, whorls were found mostly in the ring and little fingers of the right hand, and this result was statistically significant. We found that the fingertip sample types of the patients involved in our study were similar to those of the control group. The ulnar loop was the most frequently seen sample type in both the right and left hands in both groups. However, when compared to healthy control groups, there was a statistically significant increase in the rate of incidence of radial loops in MS patients’ left hands.

This study is limited due to the small size of the patient group. Future studies should investigate further parameters to provide supporting evidence for genetic predisposition in MS patients. For example, dermal samples can be analyzed in the plantar region.

In conclusion, the deviation in the distribution of dermatoglyphic samples was not diagnostic; however, it supports the genetic predisposition theory in the etiology of MS. Individuals susceptibility to MS in society might be determined by analyzing dermatoglyphic samples, and the at risk group should avoid situations and environments that can trigger their disease.
